# Diet as a Potential Moderator for Genome Stability and Immune Response in Pediatric Leukemia

**DOI:** 10.3390/cancers13030413

**Published:** 2021-01-22

**Authors:** Shanshan Wang, Christopher A. Maxwell, Neha M. Akella

**Affiliations:** 1 Department of Pediatrics, Faculty of Medicine, University of British Columbia, Vancouver, BC V6H 3V4, Canada; shanshan.wang@bcchr.ca; 2 Michael Cuccione Childhood Cancer Research Program, BC Children’s Hospital, Vancouver, BC V5Z 4H4, Canada

**Keywords:** diet, pediatric leukemia, genome, immune response

## Abstract

**Simple Summary:**

Pediatric acute lymphoblastic leukemia (ALL) is the most prevalent cancer affecting children in developed societies. Here, we review the role of diet in control of the incidence and progression of childhood ALL. Prenatally, ALL risk is associated with higher birthweights of newborns, suggesting that ALL begins to evolve in-utero. Indeed, maternal diet influences the fetal genome and immune development. Postnatally, breastfeeding associates with decreased risk of ALL development. Finally, for the ALL-affected child, certain dietary regimens that impact the hormonal environment may impede disease progression. Improved understanding of the dietary regulation of hormones and immunity may inform better approaches to predict, protect, and ultimately save children afflicted with pediatric leukemia.

**Abstract:**

Pediatric leukemias are the most prevalent cancers affecting children in developed societies, with childhood acute lymphoblastic leukemia (ALL) being the most common subtype. As diet is a likely modulator of many diseases, this review focuses on the potential for diet to influence the incidence and progression of childhood ALL. In particular, the potential effect of diets on genome stability and immunity during the prenatal and postnatal stages of early childhood development are discussed. Maternal diet plays an integral role in shaping the bodily composition of the newborn, and thus may influence fetal genome stability and immune system development. Indeed, higher birth weights of newborns are associated with increased risk of ALL, which suggests in-utero biology may shape the evolution of preleukemic clones. Postnatally, the ingestion of maternal breastmilk both nourishes the infant, and provides essential components that strengthen and educate the developing immune system. Consistently, breast-feeding associates with decreased risk of ALL development. For children already suffering from ALL, certain dietary regimens have been proposed. These regimens, which have been validated in both animals and humans, alter the internal hormonal environment. Thus, hormonal regulation by diet may shape childhood metabolism and immunity in a manner that is detrimental to the evolution or expansion of preleukemic and leukemic ALL clones.

## 1. Introduction

Diet and nutrition are the most critical determinants of human cancer risk [1]. In 1981, a landmark report estimated that one-third of cancers are attributable to diet [2]. Since that time, many investigations have linked improper nutrition with the process of tumorigenesis, including epidemiology observations, clinical trials, animal research, molecular mechanism studies, and genetic and epigenetic research [3,4,5]. Unsurprisingly, these associations also exist in childhood tissues.

Pediatric leukemias are blood cancers arising in children that involve the abnormal development of leukocytes, which then excessively accumulate in the bone marrow and blood and cause a host of pathologies. The most common leukemias in children are acute lymphoblastic leukemia (ALL), acute myeloid leukemia (AML), chronic lymphocytic leukemia (CLL), and chronic myeloid leukemia (CML). The incidence for each of these diseases is quite low, but pediatric ALL is by far the most prevalent, making up approximately 75% of all childhood leukemia patients [6]. Indeed, pediatric ALL is a leading cause of non-accidental deaths among children in developed countries [7]. Fortunately, patients suffering from pediatric leukemia have high cure rates. However, the incidence of the disease has been consistently increasing, and it is proposed that a combination of prenatal and postnatal factors contribute to this increasing risk [8]. This review will discuss the associations between diet and nutrition with childhood leukemia risk and prognosis, the maintenance of genome stability, the generation of immune responses, and the management or surveillance of preleukemic and leukemic clones.

## 2. Associations between Nutrition and the Development of Childhood Leukemia

Some childhood cancers have embryonic or pre-natal origins. In B-cell acute lymphoblastic leukemia (B-ALL), for example, pre-cancerous embryonic cells exist prenatally, and their survival and ability to initiate tumorigenesis depend on genetic events and acquisition of mutational load [9]. Primitive fetal hematopoiesis begins in the yolk sac, followed by definitive hematopoiesis (fetal and adult lineages) in the aorta-gonad-mesonephros region [10], before localizing to the fetal liver prenatally and bone marrow postnatally [11]. The first oncogenic event leading to partial transformation in-utero is proposed to occur as development progresses through these stages [11]. Transformation and cell susceptibility are attributed to developmental errors, cell-intrinsic factors, and cell-extrinsic factors in the environment, including maternal regulation of fetal development [11]. The fetus is affected by subtle changes in maternal nutrition with effects that carry over to adult life. Thus, maternal health is crucial to fetal development and the regulation of developmental origins of disease and disease predisposition [12].

The connections between diet and disease are extremely complex, and variations in study designs and research subjects may explain conflicting associations. However, some dietary associations with risk of developing childhood leukemia are now well-defined, including a balanced maternal diet. Regular consumption of vegetables and fruits/fruit juices by mothers during pregnancy [13,14] and by children up to the age of two, could reduce the risk of leukemia development between 2 and 14 years of age [15]. In a study on the Han Chinese population, a diet rich in bean curds and vegetables was protective, whereas cured/smoked meat was associated with increased risk [16]. A similar increase in risk was associated with a maternal diet rich in sugars, syrups, and meats, and a decreased risk with consumption of vegetables, fruits, legumes, and seafood [13,17,18]. Surprisingly, these associations can be made with diet quality up to a year before pregnancy. That is, an overall healthy diet in women prior to pregnancy could lower the risk of leukemia in offspring; there is a stronger association with children diagnosed before the age of five [19].

Folate supplementation and low maternal intake of alcohol and caffeine are also associated with lower risk for childhood leukemia. Adequate folic acid and other micronutrient supplementation during pregnancy is associated with lower risk of childhood leukemia, over various populations and backgrounds, possibly through epigenetic modifications that drive oncogenesis [17,20,21]. Conversely, maternal alcohol intake both during and prior to pregnancy can increase the risk of childhood leukemia, and the risk increases even further with increased frequency of alcohol intake [22,23]. A comprehensive analysis that combined reports from multiple case studies reported a potential adverse effect of consuming more than a cup of coffee a day in a dose response manner during pregnancy [24]. Previous meta-analysis of over 3500 cases, observed a correlation between childhood leukemia and high coffee and any cola consumption in pregnant mothers, and inversely, a protective effect of tea consumption [25]. No such associations with tea consumption were made in the more recent analysis [24]. Although the analysis did not go into specifics of causation, it alluded to probable mechanisms from previous studies—caffeine has been known to cause inhibition of ATM kinase [26], tumor suppressor p53 [27], and DNA topoisomerase II [28], all of which play roles in leukemia [24]. Topoisomerase II inhibition through maternal dietary sources in close to 100 mothers was significantly associated with increased AML risk in infants with a mixed lineage leukemia (MLL) gene translocation [29]. Although the associations with alcohol, caffeine, and smoking are most frequently observed in childhood ALL, they are also seen in other types of childhood leukemia, lymphoma, Wilms’ tumor, and brain cancer with transplacental exposures to these factors [30].

Breastfeeding has been promoted for its protective health benefits, access, and low-cost impact. Multiple studies measured childhood leukemia outcome in response to breastfeeding exposure, summarized in a meta-analysis by Amitay and Keinan-Boker [31]. Using stringent selection criteria to narrow down on high quality studies, 17 different case studies conducted between 1960 and 2014 were analyzed. Breastfeeding for more than 6 months was found to be protective and decreased risk of leukemia by 20%, and breastfeeding ever compared to no breastfeeding at all was enough to lower risk by 9% [31]. Breastmilk is rich in maternal antibodies, maternal cells (breast-derived early-stage stem/progenitor cells and blood-derived immune and hematopoietic cells), anti-inflammatory molecules, and enzymes [32]. Maternal cells and signaling molecules in breastmilk have been found to survive long term in the offspring, enabling them to reach various organs and tissues and imparting immunity and a healthy gut microbiome to infants, thereby offering protection against diseases. Breastmilk constituents are known to modulate immune regulation, tolerance, tissue-repair, and overall infant development [32,33]. Thus, it would be fair to hypothesize that breastmilk also impacts disease susceptibility, especially in a disease of the immune system, such as leukemia. Indeed, a recent metabolomics analysis on neonatal blood spots found that patients that went on to develop early (ages 1–5) and late (ages 6–14) leukemia, had distinct metabolomic profiles [34]. The study revealed that the presence of certain fatty acids, several of which were related to breastmilk, breastfeeding duration (reduced risk), and maternal body mass index (BMI) (increased risk), could influence risk of early and late leukemia incidence [34].

It is also worth considering environmental exposures to chemicals indirectly through food consumption or packaging, such as pesticides, solvents, and other pollutants [35]. A meta-analysis by Heindel et.al. [36] summarizes 425 epidemiological studies between 1988 to 2014 that report disease outcomes correlated with exposure to environmental pollutants. The most common exposures were to industrial chemical polychlorinated biphenyls, organochlorine pesticides, mercury, and lead [36]. Health outcomes of neurological deficits and cancer were among the highest. Interestingly, most of the reported exposures occur in-utero and/or during early development. Fetal exposure to chemicals has been linked to leukemia diagnosis outcomes in infancy and childhood [36]. While associations with maternal exposures are more frequently reported, there is also an association between paternal occupational exposure to pesticides and other industrial solvents to increased childhood cancer risk [37]. In addition to chemical exposures, exposure to low dose radiation has potential long-term health effects. Nuclear workers exposed to low dose radiation over a long period of time, children exposed to natural background radiation in-utero and infancy, and those exposed to medical imaging technology, all point to some degree of association with increased cancer incidence [38,39,40]. In fact, leukemia was the most frequently reported cancer among the survivors of the atomic bomb, with a much higher risk in children under the age of 10 [41]. This is not surprising, given that bone marrow is highly sensitive to ionizing radiation, which then causes damage to hematopoietic stem cells [42]. Finally, other environmental cues such as stress can also induce epigenetic modifications in-utero that are responsible for development of adult health defects and disease [43].

**Figure 1:**
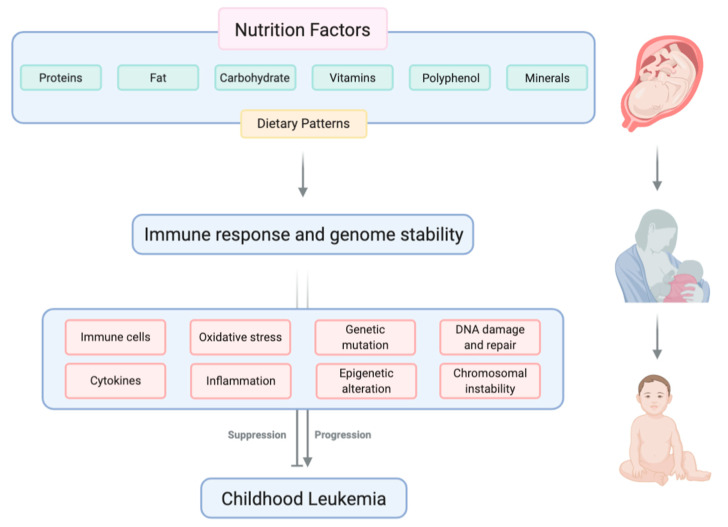
Nutritional factors, including macronutrients and micronutrients, can influence the development of childhood leukemia by modulating immune response and genome stability. Created with BioRender.com.

Adult and childhood cancers are frequently characterized as genetic diseases. While the connection between genetic mutations and cancer is unquestionable, it is equally clear that the expression and consequences of harmful or pathogenic mutations can exist on a continuum that can be modified and overseen by the tumor or tissue microenvironment. Additionally, while genetic mutations may be heritable and hard-wired, the tumor or tissue microenvironment is likely to be more pliable and responsive to exogenous factors, such as diet and nutrition. It is, therefore, worthwhile to closely examine the impact of nutrition on childhood leukemia development (Figure 1) and prognosis.

## 3. Nutrition and the Prognosis or Treatment of Childhood Leukemia

Inadequate nutrition, often due to socio-economic forces, can increase predisposition to diseases, and/or reduce survival in pediatric cancer patients. For example, malnourished patients at diagnosis had poor prognosis and overall survival rates to treatment, which worsened with time [44]. In a 2017 study in Nicaragua, about 70% of patients of ages 6 months to 18 years were malnourished with increased treatment-related morbidity and reduced event-free survival [45]. In addition, malnutrition reduced patient immunity and increased susceptibility to infections, thereby affecting treatment response and mortality [46]. Not only is malnutrition a poor prognostic factor for leukemia, but it is also a common side-effect in pediatric patients undergoing cancer treatment. Treatment-induced malnutrition can differ among pediatric patients, with some patients suffering malnutrition during treatment, but a few patients are at a higher risk of gaining weight [47]. While malnutrition is often observed in children living in low-income and middle-income countries, obesity is prevalent in high-income countries; both malnutrition and obesity are associated with poor prognosis [48].

Obesity and being overweight are among the five global risks for increased cancer incidence in the world [49]. Patient diet and nutrient intake seem to vary significantly over the course of cancer treatment, leading to changes in BMI and potentially leading to an obesogenic effect [50]. In fact, fetal embryo cord blood from obese mothers had different gene expression profiles, including changes in immune and inflammatory signaling, compared to controls [51]. In a population of about 5000 ALL patients between the ages of 2 and 20, obesity before treatment was identified to associate with high risk B-ALL, in Hispanic and male populations more than others [52]. These associations also hold in some animal models of ALL. For instance, obese mouse models of ALL displayed increased levels of insulin, leptin, and inflammatory cytokines, leading to increased oxidative stress [53].

Overnutrition or undernutrition, in addition to already varied diets among patients, can cause large fluctuations in BMI and thus in drug response [50]. It is therefore critical to develop specific nutritional plans and interventions that address toxic effects and identify essential supplementation during treatment. The European Society for Clinical Nutrition and Metabolism and the Spanish Society of Medical Oncology guidelines are two examples of nutrition recommendations developed for cancer patients. These include pre-treatment screenings; strategies for nutrition and physical activity, specialized for treatment type and stage of disease; and finally, recommendations for cancer survivors who are at higher risk for additional health conditions, especially obesity and relapse after treatment [54,55,56].

Nutrient supplementation may improve treatment outcomes for childhood leukemia. For example, glutamine supplementation was performed in ALL patients with low to moderate disease undergoing chemotherapy over four weeks [57]. Physical, biochemical, and immunological indicators were observed, and the addition of glutamine to diet improved nutritional status and reduced edema in the treatment group [57]. The overall amounts of T-cell subsets and natural killer cells significantly decreased after chemotherapy, and glutamine-fed patients had improved numbers, pointing to improved immune function [57]. Along the same lines but in an acute leukemia mouse model, two strains of lactobacillus were supplemented in diet to enhance immunity [58]. The mouse model showed loss of gut microbiome components, and reintroduction of lactobacillus reduced inflammatory cytokine production and muscle atrophy markers. However, this effect was not seen when supplemented with other strains of lactobacillus [58].

Retrospective analysis of dietary intake has also revealed associations between diet and treatment outcomes. In a dietary intake study that evaluated over 500 patients undergoing treatment, bacterial infections were found to be reduced in patients who consumed more B-carotene and vitamin A through diet [59]. Additionally, vitamins A and E, and zinc from dietary sources and B-carotene supplementation reduced the incidence of mucositis. Interestingly, patients on supplements below dietary recommended intakes had increased rates of bacterial infections and mucositis during post-induction treatment [59]. Another study observed vitamin D deficiencies associated with decreased survival and poor outcome in childhood leukemia patients in India, most of whom were deficient before treatment and some after chemotherapy [60]. Mortality during treatment induction, commonly related to infections, was high in vitamin D-deficient patients [60]. A limited small-scale study of less than 25 patients showed increased cellular oxidative stress associated with high protein consumption [61]. Correspondingly, a low protein diet in mouse models of lymphoma was able to slow tumor growth [62]. However, the majority of studies still point to benefits of proteins from good sources, such as legumes and vegetables as mentioned earlier, because childhood leukemia treatments often lead to protein malnutrition.

Proper nutrition impacts how a patient responds to treatment and should be factored in when formulating a treatment and long-term follow-up plan. In the near term, it is important to perform comprehensive screenings, and closely monitor and modify food intake of patients throughout treatment. Nutrient supplementation, for example, may modulate responses and lower treatment associated morbidities. It is clear that diet and nutrition have the potential to positively impact leukemia patients’ outcomes.

## 4. Micronutrients and Macronutrients Control Genome Stability

Foodstuffs are composed of both micronutrients and macronutrients. Micronutrients are vital in the normal functioning of life, with many acting as substrates or cofactors for enzymes responsible for genome maintenance and repair. Moderate deficiency results in genomic damage equivalent to damage caused by exposure to sizeable doses of genotoxins, such as chemical carcinogens and radiation [63]. At the cellular level, micronutrient excesses or deficiencies may lead to micronuclei formation, DNA oxidation and chromosome damage, and loss of DNA transcription fidelity, and are likely significant contributors to transformation [64,65,66]. It is prudent to highlight a few specific examples. Iron deficiency is the most common micronutrient deficiency in children worldwide. Daily iron supplementation, up to two-fold of the dietary recommended intake, can prevent micronuclei formation and enhance genome stability [67]. Iron has been recognized as an essential component of fundamental cell cycle progression [68]. Iron status variation can influence DNA metabolism functions by affecting multiple enzymes required for DNA synthesis and repair [68,69]. A recent study showed that human family B DNA polymerases required iron as a cofactor to maintain the enzyme’s function and stability to influence the fidelity of DNA synthesis [70]. However, overload of iron has also been linked to genome instability and increased cancer risk [71]. Excessive iron compounds can induce genotoxicity by increasing the mutagenic response in mouse lymphoma assays. Overdose of iron has been reported to induce double strand breaks in mouse models [72]. Thus, it is essential to elucidate the appropriate dosage of iron supplement to improve health and nutrition balance without the toxic effects.

Calcium, another essential mineral for life, is necessary for multiple biological functions, including building healthy bones, muscles, and nerves, and maintaining the fundamental cell progressions [73]. However, exposure to high calcium concentration may induce genome instability. In an Australian study, hypercalcemia has been related to the formation of shorter telomeres [74]. Hypercalcemia can also increase chromosomal damage and is linked to the incidence of childhood leukemia [75]; moreover, high plasma calcium was positively correlated with micronuclei formation in a cross-sectional study involving 462 children [76]. Similar results are reported for high intakes of magnesium and zinc and micronucleus frequency [77]. Zinc, an essential component of over 300 enzymes [78], is critical to the control of cellular growth and genome stability [79,80]. An in vitro study found that zinc deficiency or excess was tightly related to increased DNA damage and chromosome instability in human lymphocytes [81]. Micronuclei frequency and apoptotic and necrotic percentages were significantly increased in zinc deficient and zinc-oversupplied cultures [81]. Excesses in aluminum or copper can also prompt the accumulation of DNA damage [82,83]. However, another study by Fenech et al. [84] found that supplementing with vitamin E, calcium, folate, retinol, and nicotinic acid was associated in each case with a significant decrease in genomic damage, as indicated by reduced micronuclei frequency; this study also correlated an increase in micronuclei formation with high intakes of certain micronutrients, including riboflavin, pantothenic acid, and biotin. Thus, micronutrient intake is an essential requirement for the maintenance of genome stability and must be carefully regulated and controlled.

Macronutrients include dietary protein, fat, and carbohydrates, and all are indispensable in the regulation of normal physiology. Dietary protein provides amino acids, some of which the body cannot produce on its own, as a foundation upon which all other protein molecules in the body are formed. Soybeans are a critical dietary protein source in many countries. Dietary soy protein can decrease circulating insulin levels and prevent insulin-induced DNA damage [85]. Conversely, excessive protein intake, like a high casein diet, may induce DNA damage, which can be abolished by dietary-resistant starch [86]. In pediatric leukemia patients, protein consumption is thought to alter intracellular oxidative stress, and as a consequence, influence chemotherapeutic efficacy [61].

Dietary fat provides glycerides and fatty acids that are important for regulating energy storage and body temperature. Dietary fat is mainly derived from animal fats and plant fats, consisting predominantly of saturated fats and unsaturated fats, respectively [87]. In an in vitro study that treated human aortic endothelial cells with omega-3 fatty acid, researchers found supplementation with polyunsaturated omega-3 fatty acids can attenuate oxidative stress-induced DNA damage, including abasic sites, oxidized bases, and strands breaks [88]. Conversely, a high fat diet may result in DNA damage by increasing hydrogen peroxide, superoxide production, and expression of oxidative stress response genes [89,90]. In a recent prospective cohort study, children born to mothers with high BMIs were at high risk of developing leukemia [91]. A similar association of increased leukemia in overweight mothers was found in a retrospective case-control study in California [92].

Nutrition status is likely to intersect the balance between genome stability and pediatric leukemia initiation, progression, and treatment. These complex interconnections are being dissected through emerging nutrigenomics analyses, which aim to decipher nutrient–gene–cancer networks. Briefly, Table 1 outlines recent nutrigenomic studies in the field of pediatric leukemia.

## 5. The Role of Nutrition in the Developing Immune System and the Management of Preleukemic and Leukemic Clones

To date, the etiology of leukemia has been primarily attributed to an interplay between genetic mutations and the response of the developing immune system to early-life infections. This conclusion stems from a hypothesis first raised by Greaves [100]. In 1988, Greaves first addressed that early-life infection was a crucial factor in the immune response and the immune cell network [100]. Lack of infectious exposure neonatally and in infancy, for instance, when isolated in a more affluent “hygienic” environment, might fail to program the immune system. Delayed exposure to common infections, along with abnormal immune function, might result in childhood leukemia [101]. As additional evidence has emerged in recent years, Greaves’ hypothesis has been more widely accepted as a cause for leukemia development. Studies after Greaves that sought to consolidate his hypothesis looked into immune modulation of preleukemia by focusing on preleukemic children’s vaccination history, daycare attendance, common early-life infections, and the presence of autoimmune responses such as allergies. A nation-wide vaccine trial compared the early and late phase with the haemophilus influenzae type B vaccine in 11,000 children born in Finland and found early vaccinations may reduce the risk of childhood leukemia [102]. A French case-control study also supported Greaves’ hypothesis by identifying inverse associations between childhood leukemia and day-care attendance, early common infections, and breast-feeding periods [103]. Evidence of a protective role of early-life infection exposure was identified in the Northern California Childhood Leukemia Study which enrolled 699 ALL cases and 977 controls. In their study, ear infection before 6 months was associated with a reduced risk of ALL among Hispanic and non-Hispanic populations [104]. Compared to early-life (first 3 months) infection, late phase (6–9 months) exposures to influenza and respiratory syncytial virus (RSV) were strongly associated with incidence of pediatric ALL [105]. By the same token, allergies have been found to lower the risk of childhood leukemia, although they are not related to infections [106,107]. Animal evidence also supports Greaves’ hypothesis. Immunostimulatory DNA-containing unmethylated CpG dinucleotides (CpG ODN) can mimic the “danger” signal from infections provided to the immune system. In vivo treatment with CpG ODN can enhance the innate immune activity, and thus induce durable alleviation and immune-mediated protection in ALL [108].

While normal early-life infections can reduce the risk of leukemia, more serious clinically diagnosed infections can be indicators for abnormal immune function, which can increase the probability of developing the disease. More episodes of medically diagnosed infections were associated with increased incidences of both childhood ALL and AML [109]. A matched case-control study enrolling 1600 cases ALL and 16,000 controls aged 2–14 years from Canada demonstrated that having more infections (>2 infections/year) or an infection between the age of 1–1.5 years may increase the risk of developing childhood ALL [110]. In conclusion, Greaves’ hypothesis and the additional confirmatory studies show that early-life infections can “educate” the developing immune system and correct its course before developing complete childhood leukemia. However, this conclusion assumes an immune system that is robust in tackling infections.

Prenatal and postnatal development of the immune system plays a pivotal role in determining the risk of acquiring childhood leukemia. Importantly, the balance of macronutrients and micronutrients has demonstrable impacts on systemic inflammation and immunity [111,112]. Obesity, for example, is linked to chronic inflammation through a variety of mechanisms. Excessive free fatty acids released from the adipose tissue promote inflammation by binding to the Toll-like receptors, a family of transmembrane proteins that are responsible for controlling inflammatory and immunological responses, consequently increasing the expression of multiple pro-inflammatory cytokines [113,114,115]. These cytokines are essential for tumorigenesis, as they stimulate the expression of adhesion molecules on stromal and immune cells, and induce angiogenic factors, chemokines, matrix metalloprotease proteins (MMPs), and reactive oxygen species (ROS) [116,117]. Obesity is also known as a significant driver of macrophage infiltration and the dysregulation of M1/M2 macrophage balance, which influences many different aspects of tumor progression [118,119].

Micronutrient components, such as vitamin A, vitamin C, and vitamin D, also influence cancer prevention and therapy [120]. Vitamin A and its bioactive metabolite, retinoic acid, regulate immune activity [121]. Retinoic acid can enhance natural killer cell activity, IL-2 secretion, and the CD4+/CD8+ T cell ratio. For these reasons, several experimental studies and clinical trials have examined the essential role of retinoic acid in the treatment of hematological malignancies, such as cutaneous T cell lymphoma and acute promyelocytic leukemia [122,123,124,125,126]. Vitamin C, a powerful antioxidant, promotes immune defense by regulating various cell functions [127]. A recent experimental study showed that vitamin C could inhibit tumor growth by increasing the cytotoxic activity of adoptively transferred CD8+ T cells [128]. Vitamin C can also elevate the efficacy of immune checkpoint inhibitors to induce a complete response and reduce monotherapy doses [128]. Finally, vitamin D has also been considered to have an anti-proliferation effect in melanoma by strengthening cytotoxic activity and functioning as an immune regulator through the expression of immune checkpoint modulator PDL-1 [129]. While there is much to learn, it is clear that nutrition may have significant beneficial effects on immune activities, including responses to early-life infection and the surveillance of preleukemic and leukemic clones.

## 6. Future Perspectives and Applications

Nutrigenomic studies offer insights into the mechanics of nutrient–gene interactions for preventive strategies, treatment strategies, palliative care, and to improve public health in general [130]. It is evident that nutritional and environmental exposures in-utero greatly influence childhood leukemia incidence [36]. Therefore, adequate nutrition should be promoted early as a preventive lifestyle rather than a cure. Malnutrition and obesity can also affect how the body responds to treatment, and if not managed well, can contribute to relapse. Indeed, studies highlight the need for nutritional interventions during cancer treatment to improve prognosis and quality of life post-treatment [131]. The design of clinically relevant interventions, however, is complicated by diverse variables (genetics, environment, culture, economics, intake amount, metabolic ability, lifestyle) that may heavily influence data interpretation and outcomes [132]. Moreover, changing nutritional signatures during treatment pose an additional challenge that prompts further evaluation [133]. However, the potential benefits associated with nutritional counselling and better follow-up may include the improvement of quality of life and relapse-free survival. Thus, future avenues of research should include multiple omics analysis that study the impact of nutrition on epigenetics (especially DNA damage to the genome), the gut microbiome, and other factors that may influence an individual’s dietary preferences or response to treatment [130,131,132]. For example, a recent study used a big data framework approach to predict outcomes in childhood leukemia patients [134]. Moreover, while additional animal and human studies remain a critical need in the area of nutrition, it may be useful to focus on better structured analyses, possibly incorporating machine learning, to summarize existing associations. However, in the near term, encouraging healthy eating and increasing access to affordable organic and healthy foods, which today are out-of-reach for far too many, has the potential to improve treatment responses and children’s health.

## 7. Conclusions

Childhood leukemias have an in-utero origin wherein preleukemic cells exist prenatally. Indeed, the etiology of leukemia is thought to rely on the interactions of transforming mutations occurring in immature immune cells and the developing immune system, which itself is shaped by the balance of macronutrients and micronutrients. Maternal diet and nutrition, therefore, are likely to be essential in the determination of disease predisposition. Consistently, many aspects of the maternal diet associate with a lower risk of developing leukemia, including a balanced maternal diet, folate supplementation, restricted exposure to alcohol, smoking, and caffeine. Similarly, exposures to environmental toxins, both chemical and radiation, have long-term health effects, including an elevated predisposition to cancer. Moreover, obesity and malnutrition in the affected child associate with high risk of disease, increased treatment-related morbidity, and reduced event-free survival. The molecular mechanisms that account for these adverse events are being uncovered, and include strong connections between micronutrient deficiencies and elevated occurrences of genome instability measured at the cellular level. Tragically, inadequate nutrition is often a result of socio-economic forces, but diet quality improvement may help to reduce the burden of childhood leukemia and the long-term adverse health effects of current treatments.

## Figures and Tables

**Table 1:** Overview of recent nutrigenomic studies in the field of pediatric leukemia.

Author	Objective	Methods	Results
Timms et al., 2019 [93]	Investigate the potential mediating mechanism between environment and pediatric ALL disease risk.	Epigenome-wide association study (EWAS) (n = 861–927)	DNA methylation may play an intermediary role in the causal pathway linking childhood ALL risk exposures with disease risk. Hypergeometric probability tests demonstrate directionally concordant gene methylation changes observed in ALL disease and in response to alcohol intake (*p* = 0.006); sugary caffeinated drink intake during pregnancy (*p* = 0.045).
Schraw et al., 2018 [94]	Investigate the relationship between maternal genotypes in folate-related genes and pediatric ALL.	Genome-wide DNA methylation profiling and single nucleotide polymorphism selection and genotyping method. (sample from 51 pediatric ALL patient-mother pairs and 6 healthy donors)	Maternal folate metabolism may impact leukemogenesis via DNA methylation. Differential DNA hypermethylation in patients with ALL according to maternal *MTR* genotype. Maternal *MTR* rs12759827 may influence DNA hypermethylation in ALL.
Evans et al., 2014 [95]	Identify the relative contribution of gene-environment interactions to pediatric ALL.	Using genome-wide genotyping to investigate gene-environment interaction. (358 childhood ALL cases and 1192 population controls)	*IKZF1* and *ARID5B* variants are associated with childhood ALL in an Australian Caucasian population. *IKZF1* variant is a genetic effect if the mother took folic acid or drank alcohol.
Løhmann et al., 2019 [96]	Identify associations between pre-therapeutic BMI and outcome in pediatric AML.	Multinational study of 867 pediatric AML patients diagnosed within the last two decades.	Obesity is associated with developing leukemic cytogenetic abnormalities. Pediatric AML cytogenetics appear to differ by BMI status. BMI standard deviation score associated with frequency of inv(16)/t(16;16) and t(8;21)
Potter et al., 2018 [97]	Investigate the relationship between maternal folate and related B-vitamin intake during pregnancy and pediatric ALL risk.	Pyrosequencing to confirm the target hypermethylation genes; identify directional methylation change in response to folate depletion and in ALL.	Hypermethylation of *ASCL2, KCNA1, SH3GL3, SRD5A2* in ALL is confirmed by measuring 20 patient samples. *SH3GL3* methylation is inversely related to maternal red blood cell folate concentrations. *ASCL2* methylation is inversely related to infant vitamin B12 levels.
Spector et al., 2005 [98]	Confirm maternal consumption of dietary DNAt2 inhibitors during pregnancy would increase the risk of infant leukemia.	Case-control study (240 cases of infant acute leukemia and 255 random digit dialed controls)	Fresh vegetables and fruits consumption during pregnancy may decrease the risk of infant leukemia, particularly MLL+. Maternal consumption of dietary DNAt2 inhibitors (fruits and vegetables) may increase risk of AML (MLL+) infant leukemia by inducing DNA cleavage and t(4;11) translocation.
Pimentel-Gutiérrez et al., 2016 [99]	Investigate the anti-cancer effect of curcumin on a human ALL cell line.	REH ALL cell line. Cell viability, gene expression and activation of NF-κB and caspase 3 has been detected.	Curcumin enhanced caspase-3 activation and downregulated NF-κB activation. Curcumin prevents DNA oxidation by decreasing in DNA adduct formation.

Abbreviations used in Table 1: ALL, acute lymphoblastic leukemia; AML, acute myeloid leukemia; ARID5B, AT-rich interaction domain 5B; ASCK2, achaete-scute complex homolog; BMI, body mass index; DNA, deoxyribonucleic acid; DNAt2, DNA topoisomerase II; EWAS, epigenome-wide association study; IKZF1, IKAROS family zinc finger 1; KCNA1, potassium voltage-gated channel subfamily A member 1; MTR, 5-methyltetrahydrofolatehomocysteine methyltransferase; SH3GL3, SH3 domain containing GRB2-like 3; SRD5A2, 3-oxo-5α-steroid 4-dehydrogenase 2.

## Methodology

General keywords—maternal nutrition, childhood nutrition, obesity, diet and leukemia incidence, genome stability, immune system, early-life infections, prevalence, risk, progression —were searched on PubMed and relevant results were selected for discussion.

## Abbreviations

ALL	acute lymphoblastic leukemia
AML	acute myeloid leukemia
ARID5B	AT-rich interaction domain 5B
ASCL2	achaete-scute complex homolog 2
ATM	ataxia telangiectasia mutated
B-ALL	B-cell acute lymphoblastic leukemia
BMI	body mass index
C1qG	complement component 1q
CLL	chronic lymphocytic leukemia
CML	chronic myeloid leukemia
DNAt2	DNA topoisomerase II
EWAS	Epigenome-wide association study
IL	interleukin
IKZF1	IKAROS family zinc finger 1
JNK	c-Jun N-terminal kinase
KCNA1	potassium voltage-gated channel subfamily A member 1
MLL	mixed lineage leukemia
MMP	matrix metallopeptidase/metalloproteinase protein
MN	micronuclei
MTR	maternal 5-methyltetrahydrofolatehomocysteine methyltransferase
NF-κB	nuclear factor kappa B
RA	retinoic acid
ROS	reactive oxygen species
RSV	respiratory syncytial virus
SH3GL3	SH3 domain containing GRB2 like 3
SRD5A2	3-oxo-5α-steroid 4-dehydrogenase 2
TNF	tumor necrosis factor
